# Enhancing Emergency Medicine Resident Transitions: The Impact of Structured Orientation Programs on Comfort and Preparedness

**DOI:** 10.1002/aet2.70080

**Published:** 2025-07-23

**Authors:** Jessica Baez, Erin McDonough

**Affiliations:** ^1^ Department of Emergency Medicine University of Cincinnati Cincinnati Ohio USA

## Abstract

**Background:**

Emergency medicine (EM) training programs vary in how residents are exposed to clinical responsibilities. Transitioning to new roles often causes stress and uncertainty. Structured orientation programs may alleviate these challenges.

**Methods:**

We implemented three role‐specific orientation sessions targeting post‐graduate year (PGY)‐2, PGY‐3, and PGY‐4 EM residents at a 4‐year residency program. Pre‐ and post‐surveys measured comfort with clinical, supervisory, and system‐based skills. Paired *t*‐tests assessed changes.

**Results:**

All three orientation sessions significantly improved self‐reported comfort across various domains. Participating PGY‐2 residents (*n* = 10) showed increased preparedness for managing critical conditions. PGY‐3 residents (*n* = 6) demonstrated improved confidence in procedural supervision and knowledge of rare procedures. PGY‐4 residents (*n* = 11) transitioning to attending roles showed the largest gains, particularly in billing and medico‐legal domains.

**Conclusion:**

Structured orientation improves EM resident readiness for role transitions. These findings support the integration of targeted educational interventions to enhance performance and preparedness.

## 1 Need for Innovation

Emergency medicine (EM) residency programs vary widely in how they structure clinical training. Some immerse residents in all aspects of patient care from the outset, whereas others adopt a more graduated approach that increases autonomy and responsibility over time. The Accreditation Council for Graduate Medical Education (ACGME) advises programs to provide progressive responsibility for patient care based on the learner's capabilities [1]. Regardless of the specific model of residency curriculum, there is broad consensus that residents' clinical, procedural, and leadership skills evolve significantly throughout training, requiring educational strategies that align with their developmental stage. Transitions between these stages, such as moving from junior to senior resident or from trainee to independent practitioner, represent critical moments in a resident's professional growth and identity formation [2, 3, 4, 5].

These transitions can be fraught with uncertainty, increased stress, and diminished confidence, particularly as residents take on new supervisory or leadership responsibilities [3, 6]. Such transitions are complex, shaped by organizational, interpersonal, and personal factors that often require rapid adaptation [6]. One study has begun to address these challenges through the development of a life skill curriculum aimed at supporting emergency medicine residents' transition to independent practice [7], but there remains a notable gap in the literature. Few studies offer guidance on how to systematically design, implement, or evaluate educational interventions tailored to intra‐residency role transitions [7, 8].

## 2 Background

Structured orientation programs offer a potential solution to bridge these transitions effectively. Prior research has demonstrated that such programs can improve preparedness, support identity formation, and promote both professional and personal development [5, 9, 10]. For example, initiatives such as transition‐to‐practice curricula in EM have highlighted the value of targeted training on life skills, supervision strategies, and medico‐legal responsibilities [7].

The transition from medical student to EM intern has been relatively well‐studied, with multiple programs describing structured approaches to onboarding and intern orientation. Qualitative work has explored the emotional landscape of this transition, highlighting common themes of anxiety, anticipation, and identity shift among incoming EM interns [11]. Educational interventions have also been developed to support this transition, including flipped‐classroom models for intern orientation and national surveys examining how residency programs structure their early training to prepare new interns for clinical work [12, 13]. These efforts reflect a growing recognition of the unique challenges faced by learners entering EM and a corresponding emphasis on curricular innovation to promote confidence, competence, and well‐being during the early stages of residency. In contrast, structured support for transitions later in training, such as assuming senior resident responsibilities or entering independent practice, has received comparatively less attention and remains an important area for continued curricular development and scholarship.

## 3 Objective of Innovation

This study aimed to assess the effectiveness of these structured, role‐specific orientation sessions for EM residents transitioning into key roles: from proceduralist to resuscitation leader (PGY‐2), from resuscitation leader to supervisor of junior learners (PGY‐3), and from senior resident to attending physician (PGY‐4). By evaluating residents' self‐reported comfort and preparedness before and after these targeted interventions, we aim to generate evidence to inform best practices in graduate medical education and to support safer, more confident transitions across the EM training continuum.

## 4 Development Process

This was a single‐site, pre‐/postinterventional study conducted at a single academic medical center with a 4‐year residency training program and Level 1 trauma center designation. This study was approved by the Institutional Review Board (IRB) under an umbrella IRB education protocol [14]. Eligible participants were PGY‐2 through PGY‐4 residents scheduled to transition to new clinical roles. Participation was voluntary, and although efforts were made to release residents from clinical duties, scheduling conflicts such as vacations and global health electives occasionally limited full participation.

The University of Cincinnati Emergency Medicine Residency Program follows a graduated responsibility model, with residents assuming increasing levels of autonomy and leadership across each post‐graduate year. PGY‐1 residents are primarily responsible for direct patient care across a range of acuities and typically staff cases with PGY‐4 residents, who function as supervisory “junior attendings” overseeing large portions of the emergency department (ED). PGY‐2 residents manage their own area of the ED, staff directly with attending physicians, and serve as the primary proceduralists for the ED. PGY‐3 residents lead resuscitations and are responsible for supervising care in the critical care area, in addition to managing a cohort of standard ED beds. In 2019, feedback from our internal annual program evaluation highlighted resident concerns about the stress and uncertainty associated with these role transitions and called for more structured support. In response, the Residency Leadership Team (RLT) implemented orientation sessions for rising PGY‐3 and PGY‐4 residents, with curricula designed to address perceived gaps in comfort and preparedness. The structure and content of the sessions were created by the RLT, creating the learning objectives by using specific feedback from the program evaluation. These sessions were informed by ongoing resident feedback rather than a formal needs assessment and have evolved iteratively over time through annual cycles of evaluation and refinement. As new technologies and treatments such as transesophageal echocardiography, extracorporeal cardiopulmonary resuscitation, and advances in acute stroke care have been introduced in our ED, the clinical leaders driving these innovations have been actively involved in these orientation sessions to familiarize residents with their use and facilitate comfort with implementation in real‐time care. In 2024–2025, an orientation session for graduating residents entering attending roles was added for the first time. This session formalized previously informal billing guidance and was developed based on anecdotal feedback from alumni reflecting on the unaddressed challenges they encountered during the transition to independent practice.

Three structured orientation sessions were evaluated during the 2024–2025 academic year. R3 orientation (for PGY‐2s) included simulation training, leadership workshops, and interdisciplinary communication scenarios to help prepare rising PGY‐3 residents for the transition from primary proceduralist in the department to resuscitation leader in the critical care zones of our ED. R4 orientation (for PGY‐3s) focused on procedural supervision, case‐based simulations, and mentoring strategies to prepare rising fourth‐year residents transitioning to a role in which they primarily supervise junior residents. Attending orientation (for PGY‐4s) covered ED flow, billing and documentation skills, medico‐legal principles, and certification examination preparation for graduating PGY‐4 residents.

## 5 The Implementation Phase

Each orientation session was coordinated by a member of the RLT and involved a multidisciplinary group of educators selected for their content expertise. A total of 13 faculty and 6 senior residents participated in teaching across the 3 PGY‐level orientations. Faculty included four general EM attendings, one dual‐trained EM/neurocritical care physician, four ultrasound‐trained EM faculty, one Emergency Medical Services (EMS) faculty member, one community EM physician practicing in an RVU‐based model, one trauma surgeon, one EM research faculty member, and two research assistants. Content‐specific sessions were led by faculty with corresponding expertise. For example, the EMS physician led pre‐hospital medical direction discussions, the trauma surgeon facilitated thoracotomy training, and the community EM physician led the billing session. Senior residents assisted in procedural skill sessions, led peer question and answer panels, and supported case‐based discussions relevant to transitioning responsibilities.

Orientation sessions were scheduled outside of clinical shifts to maximize participation. Residents scheduled on an ED or off‐service block were formally scheduled off clinical duties for the orientation day. However, some residents on vacation or away on global health electives were unable to attend. For those unable to participate in real time, session objectives and materials were shared, and hands‐on practice opportunities were offered, though none chose to pursue them. Sessions were delivered through a mix of group‐based learning (e.g., simulations, didactics, and panel discussions) and individualized practice (e.g., peer teaching in airway laboratories, certifying examination cases). Specialized spaces and equipment were mobilized, including a simulation laboratory and high‐fidelity mannequin for trauma leadership training, airway phantoms for difficult airway procedures, a subclavian line task trainer and ultrasound machine for board case simulations, a practice transesophageal echocardiography (TEE) probe and simulator for TEE training, and a cadaver with a surgical training laboratory for thoracotomy practice. All participating faculty volunteered their time, and lunch was provided for learners.

Pre‐ and post‐orientation surveys were administered on the day of each session to assess resident confidence and perceived preparedness before and after the intervention. These surveys assessed comfort using a 5‐point Likert scale. Items were tailored to each transition's competencies. Open‐ended responses were collected for the R4 and attending orientation sessions to seek qualitative feedback for future session modifications and improvement. Quantitative data were analyzed using paired *t*‐tests.

## 6 Outcomes

Resident participation and survey completion varied slightly across training levels. For the R3 (PGY‐2) orientation, 14 out of 14 residents (100%) attended and completed the pre‐orientation survey, while 10 out of 14 (71%) completed the post‐survey. At the R4 (PGY‐3) orientation, 12 out of 14 residents (86%) attended and completed the pre‐survey, with 6 out of 12 attendees (50%) completing the post‐survey. For the attending attending‐level orientation, 11 out of 14 residents (79%) attended, and all attendees (11/11; 100%) completed both pre‐ and post‐surveys.

After these structured, role‐specific orientation sessions, emergency medicine residents demonstrated significant improvements in self‐reported comfort and preparedness across all measured domains (Table 1). For PGY‐2 residents transitioning into the resuscitation leader role, mean comfort scores improved in all five assessed areas, including managing acute stroke (from 3.0 to 4.3), cardiac emergencies (from 2.79 to 3.9), and trauma (from 3.57 to 4.2). PGY‐3 orientation participants reported notable increases in confidence supervising advanced airway techniques (from 2.75 to 3.67) and in knowledge of ED thoracotomy (from 1.75 to 4.33), with similar gains noted in their ability to supervise junior and off‐service learners. Finally, PGY‐4 residents preparing for the attending physician role exhibited substantial growth in preparedness, with statistically significant increases in self‐reported comfort with critical care billing (2.27–4.36), documentation of critical care time (2.18–4.27), and continuing medical education (CME) tracking (1.73–4.18).

**TABLE 1:** Mean and media pre‐ and post‐orientation survey responses from residents participating in each transition‐specific session.

Skill	Pre‐mean (SD)	Pre‐median	Post‐mean (SD)	Post‐median	*p*
PGY‐2 orientation—transition to resuscitation leader (100% pre‐session response rate, 71% post‐session response rate)					
Telemetry phone	3.43 (0.85)	3.5	4.20 (0.63)	4	0.015
Acute stroke management	3.00 (1.04)	3	4.30 (0.67)	4	0.004
Cardiac emergencies	2.79 (1.19)	2	3.90 (0.57)	4	0.008
Traumatic emergencies	3.57 (1.22)	4	4.20 (0.63)	4	0.049
Community ED readiness	2.71 (1.27)	2	4.10 (0.88)	4	0.003
PGY‐3 orientation—transition to supervisor (100% pre‐session response rate, 50% post‐session response rate)					
Supervise advanced airways	2.75 (0.75)	3	3.67 (1.03)	4	0.027
Supervise ED interns	3.17 (0.72)	3	3.83 (0.41)	4	0.044
Supervise off‐service learners	2.42 (0.90)	2	3.50 (0.84)	4	0.036
ED thoracotomy knowledge	1.75 (0.75)	2	4.33 (0.82)	4.5	< 0.001
PGY‐4 orientation—transition out of residency (100% pre‐session response rate, 100% post‐session response rate)					
Critical care billing	2.27 (0.79)	2	4.36 (0.50)	4	< 0.001
Critical care documentation	2.18 (0.75)	2	4.27 (0.65)	4	< 0.001
ABEM examination preparedness	2.64 (0.67)	3	4.09 (0.83)	4	0.002
CME tracking	1.73 (0.79)	2	4.18 (0.60)	4	< 0.001

*Note:* Respondents rated their comfort with each listed skill using a 5‐point Likert scale (1 = completely uncomfortable, 5 = completely comfortable). Response rates reported based on number of residents present at each session.

Resident qualitative feedback for both the PGY‐3 and PGY‐4 orientation sessions was overwhelmingly positive, with many participants describing the sessions as helpful, well‐organized, and surprisingly impactful. Several PGY‐4 residents specifically expressed gratitude and encouraged the continuation of the orientation in future years, noting that the sessions exceeded expectations. One resident suggested including a dedicated section on medicolegal issues. Feedback from the PGY‐3 orientation similarly reflected overall satisfaction but included suggestions for improvement. Residents appreciated the hands‐on airway training but recommended allocating more time to case‐based discussions focused on common R4 challenges and incorporating current senior residents to share their experiences. Others suggested making the airway stations more simulation‐based and reducing the time spent on airway procedures to allow for deeper exploration of teaching and leadership topics.

## 7 Reflective Discussion

Our findings demonstrate that structured orientation programs tailored to specific residency transitions significantly improve resident comfort and perceived readiness. Across all three orientations, these improvements suggest that targeted, role‐specific training effectively enhances residents' readiness for new responsibilities, addressing key competency areas associated with each transition. These results align with prior studies suggesting orientation enhances trainee self‐efficacy and patient safety [3, 9, 10]. The observed gains in the attending cohort were especially notable. These residents faced increased anxiety about billing, documentation, and professional accountability—domains often underemphasized in traditional curricula. The large improvements support integrating these subjects in non‐clinical training before graduation. Although prior research has addressed onboarding for new residents or medical students [9, 10], our study uniquely addresses intra‐residency transitions—an overlooked but crucial phase in physician development. Notably, the greatest impact of the intervention was seen in the graduating residents, emphasizing the importance of structured support during the final transition out of residency. This finding highlights the potential generalizability of such a curriculum beyond programs with formal graduated responsibility structures. All residents, regardless of program design, must eventually transition to independent practice, and even simple, focused interventions targeting nonclinical skills can ease that shift and improve preparedness.

This study has several limitations. The small sample sizes limit the generalizability of the findings and reduce the statistical power to detect more subtle effects. Additionally, the use of self‐reported comfort and preparedness measures introduces the potential for response bias, as residents may over‐ or under‐estimate their abilities. Participation in the surveys was voluntary, which may have introduced response bias. Residents who chose to complete the surveys may have had stronger opinions—either positive or negative—about the orientation sessions, potentially skewing the results. Additionally, the voluntary nature of participation contributed to variability in the number of completed evaluations across sessions and PGY levels. Finally, the study does not include objective performance‐based measures to validate the perceived improvements. However, residents' perception of their readiness for transitions may be equally important, as it can play a key role in reducing anticipatory anxiety. Future research should incorporate multicenter designs to enhance external validity, include objective evaluations of clinical performance, and explore the long‐term impact of orientation programs on resident development and patient outcomes.

Targeted orientation sessions are an effective, feasible way to prepare EM residents for critical transitions. EM residency training programs should consider adopting structured curricula tailored to key role changes to improve readiness, confidence, and ultimately, patient care outcomes.

## Author Contributions

**Jessica Baez:** conceptualization; investigation; writing – original draft; methodology; writing – review and editing; formal analysis; project administration; data curation. **Erin McDonough:** writing – review and editing; supervision.

## Conflicts of Interest

The authors declare no conflicts of interest.

## Supporting information


**Data S1.** Session objectives and outlines.

**Data S2.** Pre and post surveys.

## Data Availability

The data that support the findings of this study are available from the corresponding author upon reasonable request.
