# Supplementary material for: Enhancing Emergency Medicine Resident Transitions: The Impact of Structured Orientation Programs on Comfort and Preparedness
Source: AEM Educ Train. 2025 Jul 23;9(4):e70080. doi: 10.1002/aet2.70080 (PMC12286759; doi:10.1002/aet2.70080)
Supplement: Supplementary file 1 — Data S1. Session objectives and outlines. [file AET2-9-e70080-s001.docx]

**Enhancing Emergency Medicine Resident Transitions: The Impact of Structured Orientation Programs on Comfort and Preparedness**

Supplemental Material 1- Session Objectives and Outlines

R3 (PGY-2) Orientation Objectives

By the end of this orientation day, PGY-2 residents should be able to:

1. Recognize and manage acute ischemic stroke, including thrombolysis and thrombectomy decision-making.
2. Lead trauma resuscitations effectively, prioritizing tasks and managing consultant dynamics.
3. Describe the workflow and indications for ED extracorporeal cardiopulmonary resuscitation (eCPR) activation.
4. Balance critical care responsibilities with patient throughput in a busy ED setting.
5. Communicate clearly and assertively during high-acuity situations with strong multidisciplinary teams.
6. Deliver difficult news, such as death notifications, with empathy and professionalism.
7. Identify strategies for emotional resilience and peer support during challenging clinical moments.
8. Apply time-management and prioritization skills to lead a critical care shift efficiently.
9. Provide effective medical direction to prehospital EMS providers in emergent scenarios.
10. Reflect on the transition to senior resident roles and create a plan for continued growth.
11. Engage in meaningful discussion with PGY-3 residents to gain insight into success strategies and common challenges.

R3 Curricular Components

1. Acute Stroke Simulation and Discussion
   - Simulation of large vessel occlusion and thrombolysis cases
   - Teaching points: National Institutes of Health Stroke Scale scoring, stroke alert activation, advanced imaging interpretation, coordination with neurointervention teams
2. Trauma Leadership Simulation
   - Team-based trauma scenario with shifting priorities and conflicting consultant input
   - Teaching points: leadership skills, closed-loop communication, and assertiveness in multidisciplinary settings (especially with trauma surgery teams)
3. Critical Cardiac Emergencies and eCPR Workflow
   - Case-based discussion of cardiac arrest scenarios appropriate for eCPR
   - Teaching points: workflow mapping for activation, patient selection, and role delineation in resuscitation
4. High-Volume Community ED Strategies
   - Interactive session on time management, prioritization, and balancing patient experience and throughput with critical care responsibilities
   - Teaching points: emphasis on role modeling and expectations in fast-paced environments
5. **Prehospital Medical Direction Practice**
   - Discussion and roleplay involving common scenarios requiring EMS medical direction
   - Teaching points: communication strategies, rapid clinical decision-making, and legal considerations in providing prehospital guidance
6. Senior Resident Panel (Peer Question and Answer)
   - Facilitated discussion with current PGY-3s or recent graduates
   - Teaching points: difficult conversations (e.g., death notification), managing personal stress, navigating conflict, and adapting to increased responsibility

R4 (PGY-3) Orientation Objectives

By the end of this orientation day, PGY-3 residents should be able to:

1. Perform advanced airway techniques including cricothyrotomy, fiberoptic intubation, and intubation through supraglottic airways or airway exchange catheters.
2. Demonstrate the ability to teach procedural skills to junior learners using clear, adaptable instructional strategies.
3. Provide constructive, actionable feedback to learners at varying levels of training.
4. Apply strategies to address common challenges in clinical teaching, including disengaged or disruptive learners and limited patient availability.
5. Understand the indications, anatomy, and procedural steps involved in emergency department thoracotomy.
6. Obtain and interpret basic transesophageal echocardiographic (TEE) views and describe appropriate probe handling and care.
7. Reflect on the responsibilities of being a senior resident educator and role model in the emergency department.
8. Engage in meaningful discussion with PGY-4 residents and recent graduates to anticipate challenges and opportunities in the final year of training.

R4 (PGY-3) Curricular Components

1. Advanced Airway Skills Lab
   - Hands-on practice with difficult airway procedures: cricothyrotomy, intubation through I-gel, fiberoptic intubation, and airway exchange catheter use
   - Teaching points: procedural indications, troubleshooting, and equipment familiarity in high-stress situations
2. Peer-to-Peer Airway Teaching Exercise
   - Residents take turns guiding blindfolded colleagues through fiberoptic intubation
   - Teaching points: verbal clarity, real-time coaching, and techniques for procedural instruction
3. Clinical Teaching and Feedback Strategies
   - Case-based discussion on providing feedback to junior residents and students
   - Teaching points: frameworks for feedback, managing learner struggles, and fostering psychological safety
4. Challenging Teaching Scenarios in the ED
   - Interactive session addressing real-world dilemmas such as disengaged learners, negative team dynamics, and optimizing teaching during low patient volume
   - Teaching points: adaptive leadership, promoting engagement, and modeling professionalism
5. Emergency Thoracotomy Workshop
   - Procedural session led by trauma surgery faculty, including thoracotomy performance and decision-making discussion
   - Teaching points: anatomic landmarks, procedural steps, team coordination, and case selection
6. **TEE Overview and Practice**
   - Didactic and hands-on session covering probe handling, cleaning, and obtaining standard TEE views using a simulator
   - Teaching points: TEE anatomy, common views, probe safety, and role of TEE in emergency resuscitation
7. Senior Resident Panel (Peer Question and Answer)
   - Facilitated Q&A with current PGY-4s and recent graduates
   - Teaching points: transition to senior educator role, balancing clinical and teaching duties, and managing end-of-residency stressors

Attending (PGY-4) Orientation Objectives

By the end of this orientation day, PGY-4 residents should be able to:

1. Identify key components of critical care billing, including required documentation and common missed opportunities.
2. Apply appropriate billing codes to clinical scenarios, including critical care and procedural billing.
3. Demonstrate readiness for the emergency medicine certifying exam through structured case practice.
4. Describe the process for initial board certification, maintenance of certification, and Continuing Medical Education (CME) requirements.
5. Recognize common medico-legal issues encountered in emergency medicine and strategies to mitigate risk.
6. Reflect on the transition from resident to attending physician and anticipate early career challenges.
7. Engage in discussion with recent graduates about employment, litigation, and post-residency preparation.

Attending (PGY-4) Curricular Components

1. Critical Care Billing Didactic
   - Overview of billing principles specific to emergency department critical care
   - Teaching points: criteria for critical care time, common documentation pitfalls, and strategies to optimize reimbursement
2. Certifying Exam Case Practice
   - Residents rotate through mock certifying exam cases
   - Teaching points: clinical decision-making under pressure, structured exam format familiarity, and common pitfalls
3. Billing Game
   - Interactive, competitive group activity where residents attempt to recognize appropriate opportunities for critical care billing when given a series of patient cases
   - Teaching points: distinguishing levels of service, identifying critical care eligibility, and reinforcing documentation concepts in a fun, low-stakes environment
4. Preparing for Life After Residency
   - Didactic review of the process for board certification, maintenance of certification, and CME expectations
   - Teaching points: timelines, requirements, and practical considerations for early-career physicians
5. Recent Graduate Panel (Peer Question and Answer)
   - Open forum with recent alumni covering the transition to practice, job search, financial considerations, and litigation
   - Teaching points: medico-legal risk in EM, malpractice trends, contract negotiation, and early attending survival tips
