# Supplementary material for: Enhancing Emergency Medicine Resident Transitions: The Impact of Structured Orientation Programs on Comfort and Preparedness
Source: AEM Educ Train. 2025 Jul 23;9(4):e70080. doi: 10.1002/aet2.70080 (PMC12286759; doi:10.1002/aet2.70080)
Supplement: Supplementary file 2 — Data S2. Pre and post surveys. [file AET2-9-e70080-s002.docx]

**Enhancing Emergency Medicine Resident Transitions: The Impact of Structured Orientation Programs on Comfort and Preparedness**

Supplemental Material 2- Pre and Post Surveys

PGY-2 Orientation Pre-Survey

How prepared do you feel to be an R3?

Please respond with your current feelings about how prepared you feel prior to R3 Orientation.

* Indicates required question

1. How prepared do you feel to answer the telemetry phone?*

*Mark only one*

- Completely unprepared
- Somewhat unprepared
- Neutral
- Somewhat prepared
- Completely prepared

2. How prepared do you feel to see and manage acute stroke patients?*

*Mark only one*

- Completely unprepared
- Somewhat unprepared
- Neutral
- Somewhat prepared
- Completely prepared

3. How prepared do you feel to see and manage patients with acute cardiac emergencies?*

*Mark only one*

- Completely unprepared
- Somewhat unprepared
- Neutral
- Somewhat prepared
- Completely prepared

4. How prepared do you feel to see and manage patients with acute traumatic emergencies?*

*Mark only one*

- Completely unprepared
- Somewhat unprepared
- Neutral
- Somewhat prepared
- Completely prepared

5. How prepared do you feel to see and manage patients in the West Chester Emergency Department?*

*Mark only one*

- Completely unprepared
- Somewhat unprepared
- Neutral
- Somewhat prepared
- Completely prepared

PGY-2 Orientation Post-Survey

How prepared do you feel to be an R3?

Please respond with your current feelings about how prepared you feel post-R3 Orientation.

* Indicates required question

1. How prepared do you feel to answer the telemetry phone?*

*Mark only one*

- Completely unprepared
- Somewhat unprepared
- Neutral
- Somewhat prepared
- Completely prepared

2. How prepared do you feel to see and manage acute stroke patients?*

*Mark only one*

- Completely unprepared
- Somewhat unprepared
- Neutral
- Somewhat prepared
- Completely prepared

3. How prepared do you feel to see and manage patients with acute cardiac emergencies?*

*Mark only one*

- Completely unprepared
- Somewhat unprepared
- Neutral
- Somewhat prepared
- Completely prepared

4. How prepared do you feel to see and manage patients with acute traumatic emergencies?*

*Mark only one oval.*

- Completely unprepared
- Somewhat unprepared
- Neutral
- Somewhat prepared
- Completely prepared

5. How prepared do you feel to see and manage patients in the West Chester Emergency Department?*

*Mark only one*

- Completely unprepared
- Somewhat unprepared
- Neutral
- Somewhat prepared
- Completely prepared

PGY-3 Orientation Pre-SurveyTop of Form

R4 Orientation

Please let me know how comfortable you feel with the following.

* Indicates required question

1. How comfortable do you feel supervising advanced airway techniques?*

*Mark only one*

1. Not comfortable at all
2. Very comfortable

2. How comfortable do you feel supervising ED interns in BEF pod?*

*Mark only one*

1. Not comfortable at all
2. Very comfortable

3. How comfortable do you feel supervising off-service residents in BEF pod?*

*Mark only one*

1. Not comfortable at all
2. Very comfortable

4. How comfortable do you feel with knowledge of the steps of an ED thoracotomy?*

*Mark only one*

1. Not comfortable at all
2. Very comfortableBottom of Form

PGY-3 Orientation Post-Survey

R4 Orientation

Please let me know how comfortable you feel with the following.

* Indicates required question

1. How comfortable do you feel supervising advanced airway techniques?*

*Mark only one*

1. Not comfortable at all
2. Very comfortable

2. How comfortable do you feel supervising ED interns in BEF pod?*

*Mark only one*

1. Not comfortable at all
2. Very comfortable

3. How comfortable do you feel supervising off-service residents in BEF pod?*

*Mark only one*

1. Not comfortable at all
2. Very comfortable

4. How comfortable do you feel with knowledge of the steps of an ED thoracotomy?*

*Mark only one*

1. Not comfortable at all
2. Very comfortable

5. Do you have any suggestions on how to improve R4 orientation for the future?*

PGY-4 Orientation Pre-Survey

Attending Orientation

How prepared do you feel before this day?

* Indicates required question

1. How prepared do you feel to recognize cases appropriate for critical care billing?*

*Mark only one*

1. Not prepared at all
2. Very prepared

2. How prepared do you feel to document critical care time?*

*Mark only one*

1. Not prepared at all
2. Very prepared

3. How prepared do you feel for the ABEM Certifying exam?*

*Mark only one*

1. Not prepared at all
2. Very prepared

4. How prepared do you feel tracking your CME?*

*Mark only one*

1. Not prepared at all
2. Very prepared

PGY-4 Orientation Post-Survey

Attending Orientation

How prepared do you feel after today?

* Indicates required question

1. How prepared do you feel to recognize cases appropriate for critical care billing?*

*Mark only one*

1. Not prepared at all
2. Very prepared

2. How prepared do you feel to document critical care time?*

*Mark only one*

1. Not prepared at all
2. Very prepared

3. How prepared do you feel for the ABEM Certifying exam?*

*Mark only one*

1. Not prepared at all
2. Very prepared

4. How prepared do you feel tracking your CME?*

*Mark only one*

1. Not prepared at all
2. Very prepared

5. Any suggestions for improvement on how I can make this day better?*
